# Effect of transcranial direct current stimulation in the initial weeks post-stroke: a pilot randomized study

**DOI:** 10.31744/einstein_journal/2024AO0450

**Published:** 2024-06-05

**Authors:** Marcela Tengler Carvalho Takahashi, Joana Bisol Balardin, Paulo Rodrigo Bazán, Danielle de Sá Boasquevisque, Edson Amaro, Adriana Bastos Conforto

**Affiliations:** 1 Hospital Municipal da Vila Santa Catarina Dr. Gilson Cássia Marques de Carvalho Hospital Israelita Albert Einstein São Paulo SP Brazil Hospital Municipal da Vila Santa Catarina Dr. Gilson Cássia Marques de Carvalho ; Hospital Israelita Albert Einstein,São Paulo, SP, Brazil.; 2 Hospital Israelita Albert Einstein São Paulo SP Brazil Hospital Israelita Albert Einstein, São Paulo, SP, Brazil.; 3 Division of Neurology Population Health Research Institut McMaster University Hamilton Ontario Canada Division of Neurology, Population Health Research Institute, McMaster University, Hamilton, Ontario, Canada.

**Keywords:** Stroke, Transcranial direct current stimulation, Magnetic resonance imaging, Neurological rehabilitation, Connectome, Motor cortex

## Abstract

**Objective:**

This study aimed at assessing the alterations in upper limb motor impairment and connectivity between motor areas following the post-stroke delivery of cathodal transcranial direct current stimulation sessions.

**Methods:**

Modifications in the Fugl-Meyer Assessment scores, connectivity between the primary motor cortex of the unaffected and affected hemispheres_,_ and between the primary motor and premotor cortices of the unaffected hemisphere were compared prior to and following six sessions of cathodal transcranial direct current stimulation application in 13 patients (active = 6; sham = 7); this modality targets the primary motor cortex of the unaffected hemisphere early after a stroke.

**Results:**

Clinically relevant distinctions in Fugl-Meyer Assessment scores (≥9 points) were observed more frequently in the Sham Group than in the Active Group. Between-group differences in the alterations in Fugl-Meyer Assessment scores were not statistically significant (Mann-Whitney test, p=0.133). ROI-to-ROI correlations between the primary motor cortices of the affected and unaffected hemispheres post-therapeutically increased in 5/6 and 2/7 participants in the Active and Sham Groups, respectively. Between-group differences in modifications in connectivity between the aforementioned areas were not statistically significant. Motor performance enhancements were more frequent in the Sham Group compared to the Active Group.

**Conclusion:**

The results of this hypothesis-generating investigation suggest that heightened connectivity may not translate into early clinical benefits following a stroke and will be crucial in designing larger cohort studies to explore mechanisms underlying the impacts of this intervention. ClinicalTrials.gov Identifier: NCT02455427.

## In Brief

Castro et al. demonstrated that PD-L1 expression detected by immunohistochemistry in non-small cell lung cancer patients treated with chemoradiotherapy with or without surgery is not related to disease progression or overall survival probabilities.

## Highlights

PD-L1 expression by immunohistochemistry is positive in more than half on stage III non-small lung cancer.

PD-L1 expression by immunohistochemistry had no relationship with clinical characteristics.

PD-L1 expression by immunohistochemistry is not related to progression-free or overall survival in patients treated with chemoradiotherapy with or without surgery.

The article was produced from the Scientific Initiation carried out during the *Faculty Israelita de Ensino e Pesquisa Albert Einstein, Hospital Israelita Albert Einstein* (2017-2022).

## Graphical abstract

**Figure:**
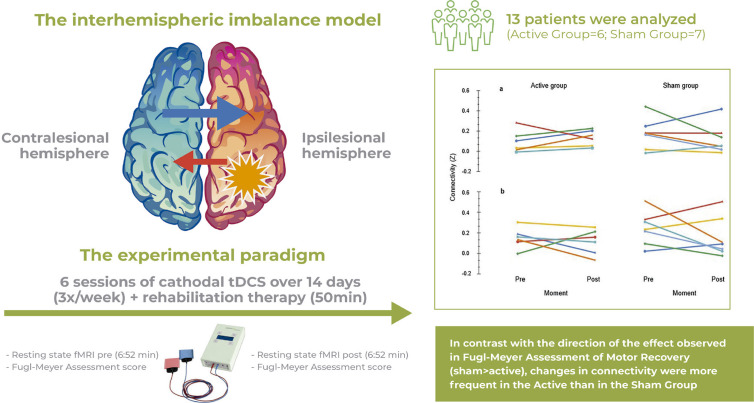

## INTRODUCTION

Stroke is the primary etiology behind long-term disability worldwide.^(1,2)^ Within a span of six months following a stroke, a predominant population of the patients, approximately two-thirds, do not experience complete recovery from upper limb paresis, which is a prevailing post-stroke impairment.^(3,4)^ Non-invasive neuromodulation interventions, such as transcranial direct current stimulation (tDCS),^(5)^ may potentiate brain plasticity and the outcomes of motor rehabilitation. Nevertheless, information regarding the effects of tDCS on the connectivity between the motor areas in the affected and unaffected hemispheres during the initial post-stroke weeks remains limited.^(6-8)^

The interhemispheric inhibition (IHI) model suggests a post-stroke imbalance in the inhibitory pathways between the two hemispheres, which strengthens the inhibitory effect on the contralesional side and prevents the recruitment of damaged ipsilesional networks.^(9)^

Protocols for tDCS conventionally employ anodal or bi-hemispheric montages. Nonetheless, experimental models have demonstrated that the application of ipsilesional anodal tDCS can potentially expand the ischemic area during the subacute post-stroke phase.^(10)^ Simultaneously, recent studies have evidenced that cathodal tDCS exhibits a contradictory effect by reducing the lesional area upon being applied ipsilesionally, which may improve the clinical prognosis of patients presenting with paresis.^(11)^

Functional Magnetic Resonance Imaging (fMRI) is capable of evaluating the impact of tDCS on brain connectivity measures. Resting-state fMRI (rs-fMRI) is particularly beneficial for patients who are unable to perform fine motor tasks owing to severe paresis.^(12)^ Rs-fMRI data^(13)^ depicted that interhemispheric connectivity between the sensorimotor cortices (SM1s) of the two hemispheres is considerably diminished in patients experiencing motor deficits in the proximate hours following a motor stroke (time of post-stroke imaging, 14±7 h) as compared to healthy individuals or stroke patients without motor deficits. On day 7, the interhemispheric connectivity pattern between the SM1s in patients exhibiting recovery tended to resemble the typical connectivity pattern observed in healthy individuals. Therefore, rs-fMRI may serve as a potential diagnostic tool that can correlate connectivity data with clinical improvements in patients.

Preliminary evidence collected at the resting stage from chronic patients with upper limb motor deficits has substantiated that clinically significant motor improvement correlates with increased connectivity between the primary motor and premotor cortices of the affected (M1_AH_) and unaffected hemispheres (PM_UH_), respectively. Overall, patients with mild deficits possess stronger connectivity between the aforementioned brain regions^(14-16)^ than severely impaired patients.

The post-therapeutic reduction in the inhibition of M1_UH_-M1_AH_ following cathodal tDCS application could enhance the connectivity of M1_UH_-M1_AH_ and PM_UH_-M1_AH_ and, consequently, serve as an alternative modality to potentiate motor recovery.

The present study advances research by evaluating the effect of cathodal tDCS using an emerging tool-rs-fMRI-in patients at an early stage after ischemic stroke.

## OBJECTIVE

The purpose of this study was to conduct a preliminary assessment of the alterations in upper limb motor impairment and connectivity between the M1_AH_ and the motor areas in the unaffected hemisphere, M1_UH_ and PM_UH_, before and after six sessions of M1_UH_, targeting cathodal transcranial direct current stimulation administered early after a stroke.

## METHODS

This pilot randomized study recruited patients enrolled in a proof-of-principle clinical trial and endeavored to use rs-fMRI to assess the safety of cathodal tDCS administered between 72 hours and 6 weeks post-stroke.^(17)^

### Study participants

Inclusion criteria were as follows: age ≥18 years; ischemic stroke confirmed by computed tomography or MRI; onset of symptoms between 72 hours and 6 weeks of the incidence of stroke; unilateral upper limb paresis quantifiable by the National Institute of Health (NIH) stroke scale (NIHSS, minimum of 1 point in items 5a or 5b, depending on the side affected by the current stroke)^(18)^ informed consent provided by the participant or proxy; and MRI performed using the same scanner.

The exclusion criteria included: stroke in the cerebellum or brainstem involving the cerebellar pathways; prior incidence of neurological disorders except migraine; history of epileptic seizure; advanced systemic disease; clinical and/or hemodynamic instability; pacemaker installation; uncontrolled arrhythmia or decompensated heart disease; modified Rankin scale score >2 prior to stroke; pregnancy; or contraindications for tDCS (scalp lesions, intracranial metal implants, and/or previous skull surgery).^(19-21)^

The sex, age, ethnicity, education, handedness,^(22)^ time post-stroke, risk factors for vascular disease, thrombolysis, lesion location (Supplementary Material, Figure 1S) and volume, and stroke etiology of patients were surveyed in accordance with the criteria of the Trial of Org 10172 in Acute Stroke Treatment,^(23)^ NIHSS,^(18)^ modified Rankin scale^(18)^ and the Fugl-Meyer Assessment of motor recovery (FMA, upper limb).^(24)^

The lesion volume was calculated after semi-automated delimitation of the infarction area. An experienced neuroradiologist classified the lesions as cortico-subcortical or subcortical and examined the involvement of the M1, PM, supplementary motor area (SMA), primary sensory cortex, centrum semiovale, internal capsule, cerebellum, and brainstem on T1-weighted and Fluid attenuated inversion recovery FLAIR images.

### Enrollment, randomization, and blinding

Participation in this study was voluntary. The participants were informed of the objectives of the research and the methodology proposed for the study. Consent forms were signed by family members on behalf of patients who verbally assented to participation but were unable to provide written consent.

The patients were recruited upon admission to our hospital and from within the community.^(25,26)^ A computer-generated blocked randomization schedule (10 blocks of 4 participants) was generated using randomization.com for allocation to either the Active or Sham Group in a 1:1 ratio.

The randomization table was secured in a locked cabinet, and password-protected files were accessible only to the investigator who administered the tDCS and the principal investigator. The patients and researchers who administered physical therapy or evaluated the outcomes were blinded to the group assignment.

This study was conducted in adherence to the principles of the Declaration of Helsinki and with approval granted by the Ethics Committee of *Hospital Israelita Albert Einstein* (CAAE: 42388115.0.0000.0071; #1.009.973).

### Experimental protocol

The experimental protocol was based on the results of a review,^(27)^ which included articles on the safety of anodal or cathodal tDCS in patients of any age with subacute or chronic stroke. The median parameters were as follows: stimulation intensity, 1 mA; duration, 20 minutes; number of sessions, five. Adverse events were assessed in 60% of studies (no adverse events, 50%). The TDCS may be administered online (during task performance) or offline (before or after task performance).^(28)^ There is no consensus on the optimal stimulation paradigm, and the timing of tDCS may have different effects according to patient characteristics and training paradigms.^(29)^ We opted for an offline paradigm, in which tDCS was delivered prior to rehabilitation therapy, which was the most widely tested paradigm at the time this study was planned.

The patients underwent six sessions of treatment over two weeks. In each session, the anode (7 × 5cm) was placed over the ipsilesional supraorbital area, and the cathode was placed in the contralesional C3/C4 position according to the EEG 10-20 reference system.^(30)^ The stimulation intensity was 1 mA. Ramping up and down lasted for 10 seconds (DC stimulator plus, Neuroconn, Germany). Transcranial current stimulation was applied for 20 minutes in the Active Group and 30 seconds, including ramping, in the Sham Group.^(31)^ After tDCS, all patients underwent 40 minutes of rehabilitation therapy.

### Outcomes

The Fugl-Meyer Assessment of motor recovery scores were conducted by blinded researchers before and after six sessions of active tDCS or sham administration. M1_AH_-M1_UH_ or M1_AH_-PM_UH_ connectivity, measured before and after treatment, was evaluated using a resting-state processing pipeline. Changes in FMA scores and connectivity after treatment were compared with baseline between the two groups. Additionally, the proportion of participants whose FMA scores met the minimal clinically important difference (MCID) after treatment relative to baseline was compared between the two groups.^(32)^

Connectivity was assessed using Fisher’s z-transform of the ROI-to-ROI correlations.

### Data acquisition

All structural and functional data were acquired using a 3T PRISMA scanner (Siemens, São Paulo, SP, Brazil). The complete MRI acquisition protocol lasted approximately 40 minutes and consisted of the following sequences: 1) Sagittal Fluid Attenuation Inversion Recovery (FLAIR): repetition time (TR) = 5000ms, inversion time (TI) = 1800ms, echo time (TE) = 386ms, time (t) = 4:12 seconds, matrix = 256 x 256, field of view (FOV) = 230mm, number of slices = 192 and voxels of 0.4 x 0.4 x 0.9mm^3^; 2) Two T1 weighted sequences, using a magnetization-prepared rapid gradient-echo sequence (MPRAGE), volumetric, 12 and 7 degree Flip Angles, with 1mm isotropic voxels, TR = 2500ms, TE = 3.5ms, FOV = 256, matrix = 256 x 256; 3) rsfMR images: TR = 2000ms, TE = 25ms, matrix = 84 x 84; FOV = 210mm, number of slices = 42 and 2.5mm isotropic voxels, with an acquisition time 6:52 minutes; and 4) for diffusion tensor imaging (DTI), EPI with isotropic voxels of 2.5mm, diffusion gradients encoded with pulses of b = 1000mm/s 2 in 36 directions and parallel acceleration (GRAPPA) with a factor of 3.

During the rs-fMRI, the subjects were asked to relax and not think of anything in particular while keeping their eyes open.

### Data processing

The lesion volume was calculated after semi-automated delimitation of the infarction area in the FLAIR images acquired using Clusterize.^(33)^ All structural images were visually inspected for artifacts (*i.e*., motion, spikes) with FSL 5.0.10 (FMRIB’s Software Library)^(34)^ by a radiologist who also determined the location of each patient’s lesions. Lesion masks were semi-automatically and manually drawn on each individual brain in the native space using Clusterize, MRIcron (https://people.cas.sc.edu/rorden/mricron/index.html), and FSL. Native-space MPRAGEs and lesion masks were warped to the Montreal Neurological Institute (MNI) space using the Clinical Toolbox Older Adult Template as the target template via a custom pipeline.

Realignment, unwarping, and slice-time correction were performed in CONN (Functional Connectivity Toolbox).^(35,36)^ Segmentation, normalization, and co-registering steps were conducted using SPM12 (Statistical Parametric Mapping; http://www.fil.ion.ucl.ac.uk/spm) and the Clinical Toolbox add-in. “Scrubbing” was performed post-realignment to identify and remove volumes acquired during periods of high motion (global-signal z-value = 5; subject motion = 0.9mm).^(37)^ Functional datasets with more than 25% of high motion volumes were excluded.

We selected six regions of interest (ROIs) from the Jülich atlas available at the FSL, based on three Brodmann areas: BA4a, BA4p, and BA6. Each of these areas had an ROI in the affected or unaffected hemisphere. These ROIs were created using a 50% probability threshold and associating the voxel with the maximum probability region of that voxel. The weights of the probability maps were used to extract the mean time series of each ROI; therefore, a spatial smoothing step was not performed. As our analysis was merely interhemispheric, the medial brain areas could be confusing as they encompass signals from both sides. Thus, the SMA was excluded,^(16)^ and areas located within 15mm of the longitudinal fissure were arbitrarily excluded. The initial ROIs were categorized into the primary motor (M1, BA4a, and BA4p) or the premotor cortices (PM, BA6), separating the affected (AH) and unaffected (UH) hemispheres. Lesions were excluded from the statistical map by overlaying the masks of individual patients on the ROIs using the command fslmaths. As an identical probabilistic atlas was applied to all participants, the starting point was the same size for all of them; consequently, the final size of each ROI was not controlled. In CONN, an ROI correlation matrix was obtained, and a Fisher z-transformation was applied to the bivariate correlation measures to prepare for the group-level general linear model (GLM). The z-scores were extracted and averaged in compliance with the desired group-level analysis (M1_AH_-M1_UH_ and M1_AH_-PM_UH_).

### Statistical analysis

Baseline characteristics were compared using one-way analysis ANOVA, χ^2^, or Mann-Whitney tests, depending on the data normality distribution analysis and the type of variable tested (quantitative or qualitative). The proportions of participants who presented with improvements in FMA scores greater than the MCID^(32)^are described. Changes in the FMA scores and connectivity (z) before and after the intervention in the Active and Sham Groups were calculated. The Mann-Whitney test was applied based on this change between groups using the software R 3.6.2 as the distribution of variables failed to satisfy the requirements of parametric tests. Statistical significance was established at p<0.05. Effect sizes were assessed using the rank biserial correlation (r_rb_)^(38)^ and calculated using JASP 0.11.1.0.

### Sample size

Considering the hypothesis-generating nature of this study, formal sample size calculations were not performed.

## RESULTS

**Figure 1:**
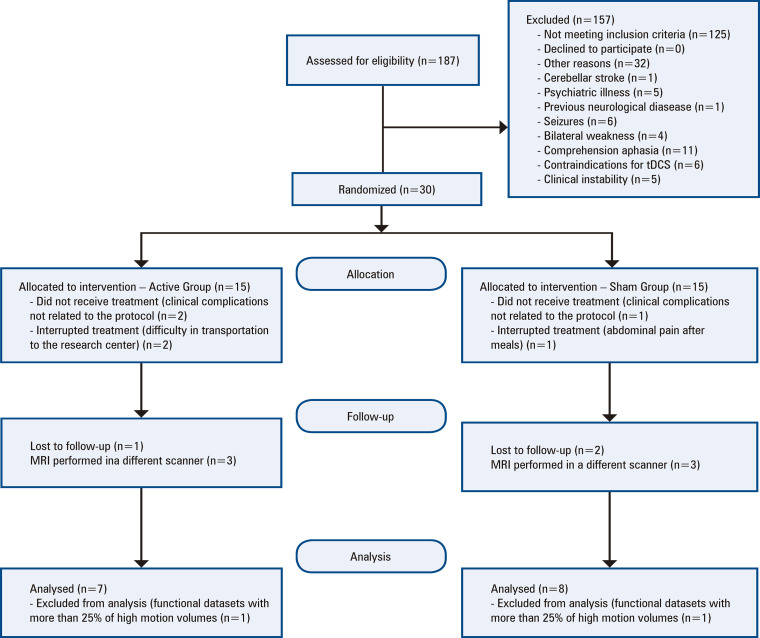
Study selection

After performing motion censoring (scrubbing) techniques to control for artifacts in the resting-state data, two patients were excluded from the analysis. Data from 13 participants were analyzed (Figure 1). There were no significant differences in the characteristics of the subjects in the Active (n=6) and Sham (n=7) Groups (Table 1).

tDCS: transcranial direct current stimulation; MRI: magnetic resonance imaging.

**Table 1:** Baseline, clinical, and imaging characteristics of the study population

Baseline characteristics	Active Group (n=6)	Sham Group (n=7)	p value
Age (years; mean±SD)	63.5±10.1	59.4±20	0.66^*^
Sex, n (%)			
Men	3 (50)	5 (71)	0.42^†^
Education (years; mean±SD)	9.33±4.41	7.7±4.46	0.52^*^
Ethnicity, n (%)	4 (66)	3 (42)	0.52^†^
White			
*Diabetes mellitus,* n (%)	2 (33)	3 (42)	0.72^†^
Yes			
Hypertension, n (%)	3 (50)	6 (85)	0.16^†^
Yes			
Handedness, n (%)	6	7	0.78^†^
Right			
Thrombolysis, n (%)	2 (33)	1 (14)	0.41^†^
Yes			
Previous stroke, n (%)	1 (16)	0	0.26^†^
Yes			
Affected hemisphere, n (%)	3 (50)	5 (71)	0.42^†^
Right			
Time post-stroke (mean±SD)	37.3±1.9	27.2±11.8	0.15^*^
NIH score (mean±SD)	6.1±4.9	6.1±3.4	0.66^§^
Fugl–meyer assessment, motor score (0-66) (mean±SD)	36.3±22.4	27.0±17.1	0.51^§^
Lesion volume (mm^3^; mean±SD)	38.2±42.3	33.0±41.9	0.83^*^
Lesion site – subcortical, n (%)	2 (33)	4 (57)	0.39^†^
Involvement of internal capsule, n (%)	4 (66)	7 (100)	0.09^†^
Yes			
Involvement of M1, n (%)	0	2 (29)	0.15^†^
Involvement of SMA, n (%)	0	0	0.78^†^
Involvement of PM, n (%)	0	0	0.78^†^
Involvement of S1, n (%)	0	0	0.78^†^
Involvement of the centrum semiovale, n (%)	3 (50)	3 (42.9)	0.79^†^
Yes			
Involvement of cerebellum, n (%)	0	0	0.78^†^
Yes			
Involvement of brainstem, n (%)	1 (16.7)	1 (14.3)	0.90^†^
Yes			
Stroke etiology – TOAST, n (%)			0.398^†^
Large-artery atherosclerosis	0	1 (14)	
Small-vessel occlusion	0	1 (14)	
Other determined etiology	1 (17)	0	
Undetermined etiology	5 (83)	5 (72)	

Baseline characteristics were compared with one-way *ANOVA; ^†^ X^2^ and ^§^ Mann-Whitney tests.

NIH: National Institute of Health; M1: primary motor cortex; SMA: supplementary motor area; PM: premotor cortex; S1: primary sensory cortex; TOAST: Trial of Org 10172 in Acute Stroke Treatment.

### FMA scores

The baseline FMA scores (0-66) were comparable between the Active and Sham Groups (Table 2). The median improvements in the FMA scores (0-66) were 14% (3%-137%) in the Active Group and 60% (5%-175%) in the Sham Group. The between-group difference was not statistically significant (Mann-Whitney tests; p=0.133; r_rb_ = −0.619). Differences ≥9 points in FMA scores (0-66)^(32)^after treatment were observed in only 2/6 patients (33%) in the Active Group and in 5/7 (71%) of the subjects in the Sham Group.

**Table 2:** FMA scores (0–66) of each subject before and after the intervention

Patients	Group	FMA Scores	

		Before	After
2	Active	59	61
3	Active	46	51
6	Active	51	60*
8	Active	46	51
10	Active	8	19*
13	Active	8	10
1	Sham	58	61
4	Sham	40	62*
5	Sham	26	42*
7	Sham	22	40*
9	Sham	8	22*
11	Sham	12	16
12	Sham	23	37*

* Increase of ≥9 points in FMA score.

FMA: Fugl–Meyer Assessment of Motor Recovery.

The patients were evaluated for motor rehabilitation in addition to the protocol, and there was no statistically significant difference between the groups (Mann-Whitney tests, p=0.484). Furthermore, descriptively, the Active Group had four more hours of physical therapy than the Placebo Group, and despite this, MCID was observed more frequently in the Placebo Group than in the Active Group.

### Rs-fMRI

The M1_AH_-M1_UH_ and M1_AH_-PM_UH_ connectivity (Mann-Whitney test; p=0.366 and p=0.234, respectively) in the Active and Sham Groups were comparable at baseline.

**Figure 2:**
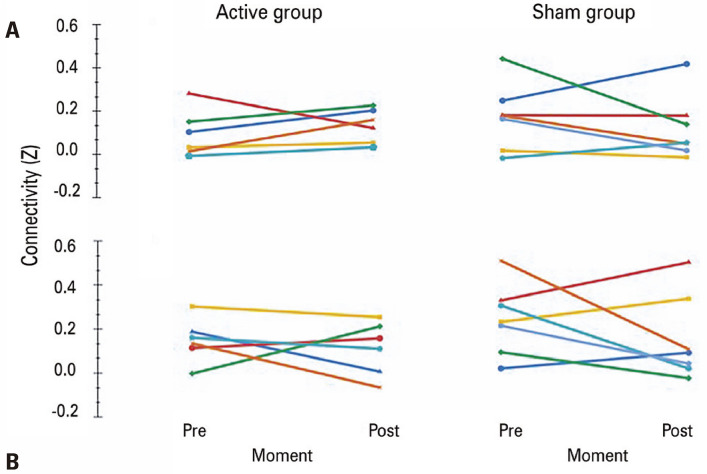
Connectivity analysis. A) Average M1AH-M1UH connectivity for each subject before and after treatment; B) Average M1AH-PMUH connectivity before and after treatment

Connectivity between the M1_AH_ and M1_UH_ increased in 5/6 subjects (83.3%) in the Active Group and in 2/7 (28.5%) subjects in the Sham Group. Changes in connectivity before and after the intervention were not significantly different between the groups (Mann-Whitney tests; p=0.295; r_rb_ = 0.381; Figure 2).

Connectivity between the M1_AH_ and PM_UH_ increased in 2/6 subjects (33.3%) in the Active Group and in 3/7 subjects (42.8%) in the Sham Group. Changes in connectivity before and after the intervention were not significantly different between the groups (Mann-Whitney tests; p=0.836; r_rb_ = 0.095; Figure 2).

## DISCUSSION

A sizeable proportion of patients in the Sham Group exhibited clinically significant enhancements in FMA scores compared to the Active Group; conversely, an elevation in M1_AH_-M1_UH_ connectivity was reported in a greater proportion of patients in the Active Group than in the Sham Group. The effect sizes were sufficient to enable modulations in the FMA scores and moderate for M1_AH_-M1_UH_ connectivity. The between-group differences were not statistically significant, presumptively owing to limitations imposed by the sample size and heterogeneity of stroke lesions.

The direction of the effect (sham > active) depicted by the FMA scores in this subgroup analysis corroborated with the findings of the principal investigation (n=30).^(39)^ A meta-analysis^(40-42)^ concerning the effects of cathodal tDCS revealed that this intervention may not be beneficial in decreasing motor impairments in early post-stroke stages.

The FMA serves as a motor impairment metric that evaluates the ability to perform particular movement synergies. The contradictory effects of tDCS on FMA and connectivity modulations indicate that distinct mechanisms underlie the impact of tDCS on behavior and resting-state connectivity. It is also possible that the resting-state outcome measures used in our study could not capture the effects of tDCS early after stroke.^(43-45)^ Furthermore, contralesional neuromodulation may potentially heighten the functional connectivity of the M1_UH_ with motor areas (for instance, SMA) that were not evaluated in this study.^(46)^

The connectivity between the premotor and primary motor regions is profoundly dynamic during the proximate weeks and months after a stroke.^(16,47,48)^ Nonetheless, the impact was insufficient to facilitate a comparison of alterations in the M1_AH_-PM_UH_ connectivity between the two experimental groups. Presumably, cathodal tDCS functions predominantly through the modulation of M1_AH_-M1_UH_ rather than M1_AH_-PM_UH_ interactions in early post-stroke stages. Alternatively, M1_AH_-M1_UH_ connectivity may lack functional relevance. The latter hypothesis is supported by prior research that investigated modifications in brain connectivity in patients suffering from upper limb paresis for over a year following a subcortical stroke.^(49)^Despite motor improvements, no statistically significant differences were recorded in M1_AH_-M1_UH_ connectivity over time. These outcomes contrast with the correlation between the restoration of connectivity and motor recovery reported by Golestani et al.^(13)^ Therefore, the relationships between brain connectivity and motor performance or recovery or the effects of neuromodulation interventions remain to be determined.

This study was constrained owing to a small sample size and inter-subject variability in FMA scores at baseline. Therefore, the results warrant cautious interpretation. Nevertheless, to the best of our knowledge, this is the first study to integrate cathodal tDCS with behavioral and rs-fMRI assessments at an early stage post-stroke. Furthermore, the study findings will be relevant for guiding future, more extensive research on the mechanisms underlying the effects of tDCS in stroke patients.

## CONCLUSION

In contrast to the effect observed in the Fugl-Meyer Assessment of motor recovery (Sham>Active), modifications in connectivity were more frequent in the Active Group than in the Sham Group. The impact of cathodal transcranial direct current stimulation on motor performance and rs-FMRI may have distinct underpinnings in patients in the early stages of stroke.
