# Supplementary material for: Effect of transcranial direct current stimulation in the initial weeks post-stroke: a pilot randomized study
Source: Einstein (Sao Paulo). 2024 Jun 5;22:eAO0450. doi: 10.31744/einstein_journal/2024AO0450 (PMC11196089; doi:10.31744/einstein_journal/2024AO0450)
Supplement: Supplementary file 1 [file 2317-6385-eins-22-eAO0450-suppl01.pdf]

## SUPPLEMENTARY MATERIAL

# Effect of transcranial direct current stimulation in the initial weeks post-stroke: a pilot randomized study

Marcela Tengler Carvalho Takahashi, Joana Bisol Balardin, Paulo Rodrigo Bazán, Danielle de Sá Boasquevisque, Edson Amaro Junior, Adriana Bastos Conforto

**DOI: 10.31744/einstein_journal/2024AO0450**

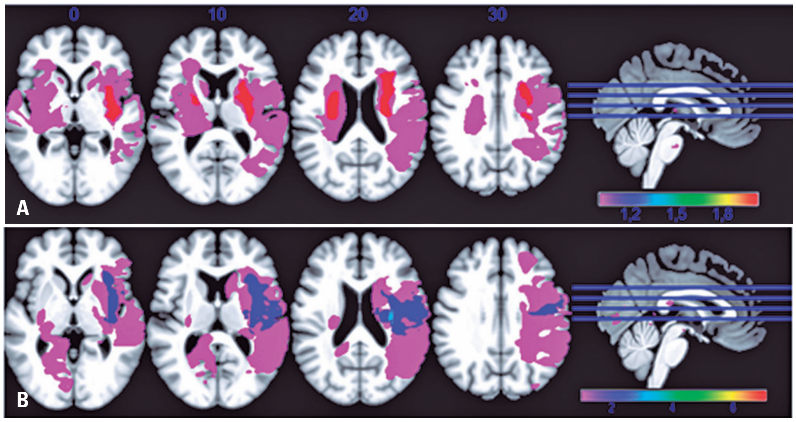


**Figure 1S.** Overlapping images of the lesions of patients of the active (A) and sham groups (B). Images recorded in the MNI atlas of the clinical toolbox. The scale indicates the degree of overlap of the lesions (red= more overlap)
